# Healthcare professionals’ perspectives towards the digitalisation of paediatric growth hormone therapies: expert panels in Italy and Korea

**DOI:** 10.3389/fendo.2024.1419667

**Published:** 2024-07-10

**Authors:** Octavio Rivera Romero, Hyun Wook Chae, Maria Felicia Faienza, Edoardo Vergani, Chong Kun Cheon, Raffaella Di Mase, Francesco Frasca, Hae Sang Lee, Claudia Giavoli, Jihyun Kim, Antonella Klain, Jung Eun Moon, Maria Laura Iezzi, James Yeh, Antonio Aversa, Young-Jun Rhie, Ekaterina Koledova

**Affiliations:** ^1^ Electronic Technology Department, Universidad de Sevilla, Seville, Spain; ^2^ Department of Pediatrics, Gangnam Severance Hospital, Yonsei University College of Medicine, Seoul, Republic of Korea; ^3^ Unit of Endocrinology and Rare Endocrine Diseases, Pediatric Hospital Giovanni XXIII, Bari, Italy; ^4^ Department of Precision and Regenerative Medicine and Ionian Area, University of Bari “A. Moro”, Bari, Italy; ^5^ Department of Translational Medicine and Surgery, Agostino Gemelli Polyclinic Foundation, IRCCS – Catholic University of the Sacred Heart, Rome, Italy; ^6^ Department of Pediatrics, Pusan National University Children’s Hospital, Yangsan, Republic of Korea; ^7^ Pediatric Endocrinology Unit, University Hospital “Federico II”, Naples, Italy; ^8^ Endocrinology Section, Department of Clinical and Experimental Medicine, Garibaldi Nesima Hospital, University of Catania, Catania, Italy; ^9^ Department of Pediatrics, Ajou University School of Medicine, Suwon, Republic of Korea; ^10^ Endocrinology Unit, Fondazione IRCCS Cà Granda Ospedale Maggiore Policlinico, Milan, Italy; ^11^ Department of Clinical Sciences and Community Health, University of Milan, Milan, Italy; ^12^ Department of Pediatrics, Dongguk University Ilsan Hospital, Goyang, Republic of Korea; ^13^ Pediatric Endocrinology Unit, Santobono Pausilipon Children’s Hospital, Naples, Italy; ^14^ Department of Pediatrics, School of Medicine, Kyungpook National University, Kyungpook National University Hospital, Daegu, Republic of Korea; ^15^ Pediatric Department, San Salvatore Hospital, University of L’Aquila, L’Aquila, Italy; ^16^ Merck Ltd., Seoul, South Korea, an affiliate of Merck KGaA, Darmstadt, Germany; ^17^ Section of Endocrinology, Department of Experimental and Clinical Medicine, Magna Graecia University of Catanzaro, Catanzaro, Italy; ^18^ Department of Pediatrics, Korea University College of Medicine, Seoul, Republic of Korea; ^19^ Global Medical Affairs, Cardiometabolic and Endocrinology, Merck Healthcare KGaA, Darmstadt, Germany

**Keywords:** adherence monitoring, Aluetta^®^Smartdot^™^, connected device, digital health, healthcare digitalisation, recombinant human growth hormone

## Abstract

**Introduction:**

To analyse the perspectives of healthcare professionals (HCPs) regarding the acceptance of digital health solutions for growth hormone (GH) deficiency care. This study identified factors impacting HCPs’ intent to use and recommend digital solutions supporting recombinant-human growth hormone (r-hGH) therapy in Italy and Korea with a use case of connected drug delivery system (Aluetta^®^ with Smartdot™) integrated in a platform for GH treatment support (the Growzen™ digital health ecosystem).

**Methods:**

Participatory workshops were conducted in Rome, Italy, and Seoul, Korea, to collect the perspectives of 22 HCPs on various predefined topics. HCPs were divided into two teams, each moderated by a facilitator. The workshops progressed in five phases: introduction of the project and experts, capturing views on the current context of digitalisation, perceived usefulness and ease of use of Aluetta^®^ with Smartdot™, exploration of the perception of health technology evolution, and combined team recommendations. Data shared by HCPs on technology acceptance were independently analysed using thematic analysis, and relevant findings were shared and validated with experts.

**Results:**

HCPs from both Italy and Korea perceived Aluetta^®^ with Smartdot™ and the Growzen™ based digital health ecosystem as user-friendly, intuitive, and easy-to-use solutions. These solutions can result in increased adherence, a cost-effective healthcare system, and medication self-management. Although technology adoption and readiness may vary across countries, it was agreed that using digital solutions tailored to the needs of users may help in data-driven clinical decisions and strengthen HCP–patient relationships.

**Conclusion:**

HCPs’ perspectives on the digitalisation in paediatric GH therapies suggested that digital solutions enable automatic, real-time injection data transmission to support adherence monitoring and evidence-based therapy, strengthen HCP–patient relationships, and empower patients throughout the GH treatment process.

## 1 Introduction

Digital health technologies and the use of connected devices are progressing rapidly, becoming an integral element of healthcare delivery (1, 2). Digital health technologies have paved the way for enhanced patient care and management of chronic conditions, especially with the advent of connected devices that facilitate the capture of objective data about patients (2). The global 5-year Easypod Connect Observational Study suggest that connected digital devices can significantly improve patient outcomes for recombinant human growth hormone (r-hGH) therapy in children with growth failure (3, 4), thereby enhancing patient adherence. They also improve therapeutic monitoring and patient support provided by healthcare professionals (HCPs) (5–7), which is a key step in the patient’s care pathway (8, 9). Adoption of digital health solutions by HCPs is correlated with desired outcomes, warranting the need to understand the attitudes of HCPs towards prescribing their use (9). HCPs remain at the forefront of creating awareness, motivating, and providing family-centered, personalised care and management; hence, adoption of digital solutions requires participatory assessment of their perceptive (5–7). Understanding factors associated with willingness of HCPs to prescribe digital health solutions to patients is important (6, 8, 10–14). However, limited information exists regarding barriers and enablers for the use of digital health ecosystems in long-term paediatric care (15).

**Figure 1:**
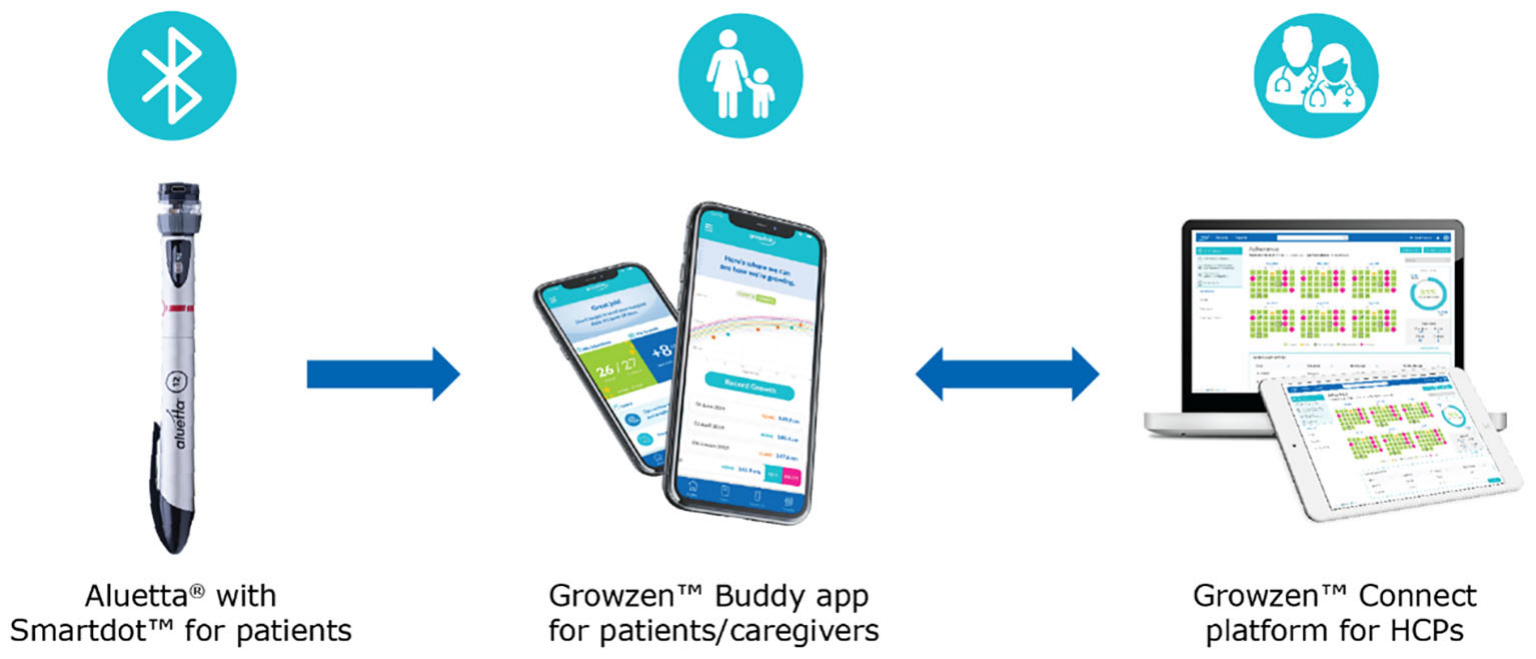
Growzen™ digital health ecosystem. Aluetta^®^ with Smartdot^™^ is used to provide growth hormone injections to patients and transmit real-time injection data. Growzen^™^ Buddy is a mobile app for patients and caregivers to guide them for growth hormone therapy. Growzen^™^ Connect healthcare professional platform is used by healthcare professionals to track treatment adherence and outcomes for growth hormone patients. HCPs, healthcare professionals.

An example of a digital health ecosystem supporting the monitoring and self-management of patients with growth hormone deficiency (GHD) is the Growzen™ digital health ecosystem. This solution includes Aluetta^®^ with Smartdot™, a novel digitally connected, reusable, multi-dose injection pen device for administering r-hGH (Saizen^®^, Merck KGaA, Darmstadt, Germany). Incorporating a smart attachment for data transmission, this innovative adherence sensor-based device combines the ease of use of the Aluetta^®^ manual pen with advanced capabilities, and its integration with a digital health ecosystem empowers HCPs with remote monitoring of patient adherence, enabling timely intervention and decision-making (16). This ecosystem currently includes Aluetta^®^ with Smartdot™, Growzen™ Buddy [a mobile app for patients and caregivers to guide them for growth hormone (GH) therapy] and Growzen™ Connect healthcare professional platform (used by HCPs to track treatment adherence and outcomes for GH patients) (**Figure 1**).

To assess the potential impact of digitalisation on the willingness of HCPs by integrating the connected GH injection pen into their clinical practice, participatory workshops involving an expert panel were conducted in Italy and Korea in 2022 to capture a broader view on acceptance of the solution across diverse healthcare ecosystems. This study aimed to understand the extent of acceptability and perceptive in countries with different level of readiness and cultural acceptance for a digital healthcare ecosystem enabled by the use of the connected injection pen for r-hGH. This qualitative study explored current attitudes towards the digitalisation of r-hGH therapy in the two countries through panel discussions, analysed HCPs’ perceptions regarding potential acceptance of the connected device compared with other non-connected alternatives (e.g., pen and paper adherence diaries), and assessed factors affecting their intent to use and integrate digital health solutions supporting r-hGH therapy in clinical practice.

## 2 Methods

### 2.1 Experts and locations

Participatory workshops were conducted in Italy and Korea to explore the perceptions of two expert panels on the acceptance of connected devices and technological evolution, considering Aluetta^®^ with Smartdot™ within the Growzen™ digital health ecosystem as an example of digital solution. The workshops were spread over 4 h and conducted on 25 November 2022 in Rome, Italy, with eight HCPs (five paediatric endocrinologists and three endocrinologists) and on 2 December 2022 in Seoul, Republic of Korea, involving 14 paediatric endocrinologists. HCPs with experience in paediatric/transition/adult GHD treatment participated in the panels regardless of their previous digital health experience. Adult endocrinologists in Italy were asked to provide their opinion on patient care focussed on the transition from patients with childhood-onset GHD to adult patients.

### 2.2 Workshop structure, activities, and materials

Experts in each workshop were grouped into two teams to independently perform several activities based on professional expertise, age, and sex. Each team was moderated by a facilitator with experience in participatory methods. The two teams were initially together in the same room for introductions and explanations of the phases and tasks of the workshop and then performed these activities in separate rooms to capture information around perceptions and then finally combined for conclusive discussions and recommendations. Data from all experts were collated for qualitative analysis.

Sticky cards representing two contexts were provided to the experts to identify factors and share their opinions according to various predefined topics. The first context considered was technology acceptance encompassing self-administration, wherein the patients administered the therapy, although caregivers took care of patients’ health and managed their treatments. The other context was the therapy administered by caregivers, wherein they took overall care of the health and therapy management with paediatric patients not being autonomous enough to be responsible for their own treatment.

**Figure 2:**
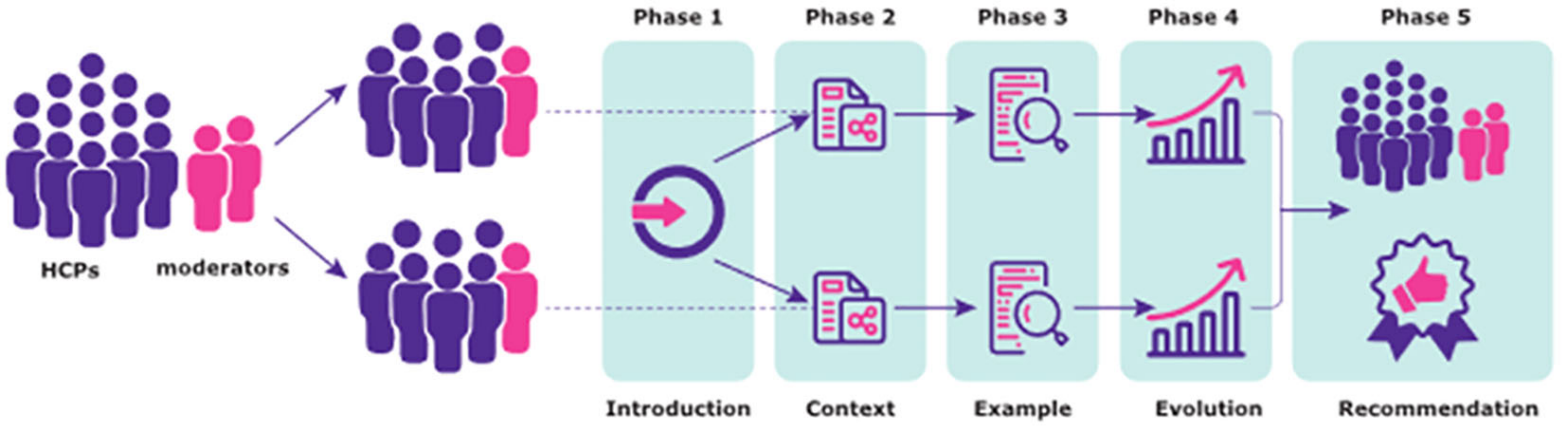
Workshop structure.

A description of Aluetta^®^ with Smartdot™ device within the Growzen™ digital health ecosystem was provided in an introductory video. Additionally, experts had the opportunity to see and touch the device during the session. During the workshop, experts were asked to provide their opinions on Aluetta^®^ with Smartdot™ orally, and the session was audio recorded to complement the notes from the moderators. Experts were prompted by various predefined topics based on their clinical experience. Each workshop progressed in five phases (**Figure 2**).

The first phase comprised an introduction of the project and experts with a description of the workshop structure and concrete tasks and activities to be performed. In the second phase, the views on the current context of digitalisation were captured, and the experts provided opinions and comments about several predefined topics such as the importance of treatment adherence, perceived usefulness of collecting patients’ adherence data, current methods used to collect adherence data, use of digital health tools with a focus on HCPs’ experience in using these solutions in their daily clinical practice, and perspectives on the patients’ attitudes towards the use of digital health tools. Experts identified factors related to three entities (patients/caregivers, healthcare centres, and HCPs) in the template and the relationship between these entities (care services, facilitating conditions, and HCP–patient relationships).

The third phase assessed the perceived utility and ease of use of Aluetta^®^ with Smartdot™ device (as an example of a digital health solution) considering both the defined contexts. Following the concrete instructions provided by the moderator, preceded by the introductory video of the device, experts discussed and identified relevant issues, strengths, and weaknesses of the digital device in the context of GHD management in their respective countries. The experts were provided with predefined study cases, namely, new pen users and Aluetta^®^ pen users (i.e., without the Smartdot™ attachment), with the lack of adherence to guide the discussions. A set of predefined topics were pursued that included relevant issues such as ergonomics, perceived ease to configure the device, perceived ease to use the device, perceived learnability, perceived ease to teach the configuration process, flexibility (removing Smartdot™), perceived usefulness for HCPs, perceived usefulness for patients, potential adoption for each study case, appropriateness for each study case, and potential risks associated with the use of the digital device.

In phases 2 and 3, predefined templates were given to the HCPs along with a set of sticky cards representing predefined topics to facilitate the activity. In the fourth phase, HCPs’ perceptions on health technology evolution in paediatric/transition/adult GHD care were explored. Three scenarios representing different technological generations were introduced by the moderator. The first scenario represented the use of a pen without any digital capability and a manual diary to collect adherence data (non-digital alternative), whereas the second scenario consisted of the use of a pen without any digital capability and a mobile app to register adherence data through manual inputs (partially digital alternative). The third scenario represented the use of Aluetta^®^ with Smartdot™ integrated in the Growzen™ digital health ecosystem to collect adherence data (fully digital alternative). Templates used in phase 3 represented these three scenarios and included topics related to the corresponding activity focussed on the adherence data collection process, potential impacts of each scenario on daily practice, and patient self-management considering both contexts. Experts were asked to identify the strengths and weaknesses of the scenarios regarding the discussed topic. Additionally, study cases defined in the previous phase were used to guide the discussions.

In the fifth and final phase, all experts were combined for team recommendations in one room. Each team briefly presented the identified factors and discussed them along with the most relevant findings on the use and recommendation of digital health solutions, in particular, Aluetta^®^ with Smartdot™ in the current healthcare setting reported during the previous activities. The moderator asked experts to describe their opinions about the relevance of factors and summarised the conclusions reached in each session.

### 2.3 Data collection and analysis

The participatory workshop sessions were audio-recorded and reviewed by the facilitators. Thereafter, relevant comments were transcribed, and information from the facilitators’ notes and text included in the predefined templates was collated. The data collected in this study were evaluated using a qualitative approach similar to that defined in the thematic analysis. Relevant findings were shared and validated with experts.

All procedures performed in this study were in accordance with European and national ethical guidelines, the European Code of Conduct for Integrity in Research, the Universal Declaration of Human Rights, and the Helsinki declaration. HCPs were informed about the research topic and procedures before joining the expert panel. The experts provided their opinions based on their experience on this topic and were not the main subjects of the study. The experts’ opinions included as quotes were pseudo-anonymised. No sensitive information was used or collected, and the contributions of the expert panel had no impact on others.

ORR reviewed all collected data, coded them, and defined themes, after which all authors reviewed the proposed themes and refined them until consensus was reached.

## 3 Results

The results presented here describe HCPs’ perspectives towards the digitalisation of growth hormone therapies and do not originate from scientific data. Overall findings suggest that each country health system and socio-economic and educational landscape may be varied, shaping the HCPs perspectives on the utility of digital health solutions and difficulty to seamlessly transfer from one setting to another. Four major themes were identified for presenting the data analysis: 1) understanding the context of digital transformation, 2) relevant digital health design considerations, 3) perceived benefits and risks of using digital solutions for adherence monitoring, and 4) perceived usefulness and ease of use of Aluetta^®^ with Smartdot™ and the Growzen™ digital health ecosystem.

### 3.1 Understanding the context of digital transformation

In this theme, experts’ comments expressing their perspectives on how their organisations were supporting the use of digital health solutions, including adherence monitoring, were recorded. Stakeholders’ perspectives on the use of novel solutions along with current strategies used to manage growth disorders were also considered.

#### 3.1.1 Organisational and technical support

This subtheme collected experts’ comments about support provided by organisations, healthcare institutions, and other entities for the use of digital health solutions towards management of growth disorders.

In both Korea and Italy, experts agreed on the importance of monitoring adherence to GH treatment. However, experts in Italy perceived that the use of digital health solutions to monitor adherence was not a priority for the Italian healthcare system due to the lack of involvement of health institutions. The potential benefits of such solutions were not considered in their evaluation. They mentioned that adherence is not often used in cost-effectiveness analysis.

In Italy, experts identified the pharmacoeconomic approach of the healthcare system while making decisions. Similarly, the Korean panel also reported some economic aspects, such as the fact that some GH treatments were financed by insurance companies that may lead to higher adherence, as suggested by academic reports. In these circumstances, monitoring adherence to treatment was mandatory, and caregivers were required to provide adherence data to physicians.

*“In case of continuous glucose monitoring it is mandatory that a certain percentage of adherence is observed in order to receive re-imbursement (insurance coverage) from the government. So, caregivers are obliged to show us the data and doctors also check it thoroughly and make data entry.” [Korea]*

While Korean HCPs could perceive the use of digital health solutions as an additional task to perform in their existing short visit time, they mentioned extrinsic motivation, such as payment of fees, as a relevant factor, whereas the Italian panel emphasised the role of the healthcare institutions in convincing and supporting HCPs in integrating these solutions (additional bureaucracy, training, and clear guidelines, etc.). Both the panels agreed on the need for training and some external services to support them in their tasks (for example, patient support programmes).

*“I would prefer a course. It’s not difficult, but I would put the need for targeted education” [Italy]*

#### 3.1.2 Perspectives on the use of a digital health solution

The Korean panel considered the Korean society to be highly digitalised and, therefore, thought that HCPs may have a positive outlook towards the use of digital health solutions. They discussed the need to tailor digital solutions to patients and caregivers and emphasised that these solutions should be user-friendly.

*“Nowadays, most people are very used to smartphones and digital applications. So, I don’t think that there will be any significant barriers in using this kind of application. But making it user-friendly, I think, is very important.” [Korea]*

Conversely, Italian experts reported that some HCPs might not be interested in the collected adherence data. Consequently, they may not promote digital health solutions due to lack of interest perceiving an increased workload and wastage of time. However, they agreed that having trustworthy data and accessing them through a usable platform would be beneficial for patient management.

*“Because this trust in having a useful device for patient management, and the attitude of trust even in regard to rapidly usable data.” [Italy]*

Both Korean and Italian panels highlighted the need to integrate digital health solutions into clinical practice. Additionally, the experts commented that this integration should be performed gradually, allowing patients/caregivers to use preferred tools for adherence monitoring. Although both panels expressed the need for actionable recommendations based on the collected data, the Korean panel preferred a brief paper summary to be used in their visits despite the highly digitalised context. However, the Italian panel agreed that they would like to receive these feedback and recommendations through a digital platform such as Growzen™ Connect HCP platform.

*“Perhaps it is not used in daily life, because there is not a moment that comes to mind during the visit to say I’m going to check this thing.” [Italy]*

Regarding patients’/caregivers’ perspectives on the use of a digital health solution to support GHD management, the Italian panel focussed on individual characteristics such as age, digital literacy, and response to external control. Although the Korean panel also identified some individual characteristics, they focussed on technical aspects. For example, they commented on the importance of encouraging patients to continue using digital health solutions for a longer duration.

Both Korean and Italian experts agreed that patients/caregivers should be aware of the potential benefits for the management of the condition of using the digital health solution. This approach was considered a key strategy to convince experts/caregivers to adopt digital health solutions.

*“What do you think about users’ attitude towards using the mobile APP? Initially reluctant, it is important to motivate them to use and explain the importance of adherence.” [Italy]*

#### 3.1.3 Managing growth disorders

Both countries reported several strategies to monitor patient adherence. Most Korean experts commented that they asked about adherence or the number of missed injections to patients or caregivers. Similarly, the current adherence data collection strategies in Italy were based on collecting subjective data, leading to unreliable datasets. HCPs also reported their concerns about the accuracy of collected data with non-digital entries.

*“It is well known that adherence is related to treatment efficacy, and most caregivers understand that their children need regular injections to be able to expect a good treatment outcome. This then leads to the question of ‘how can we improve adherence?’” [Korea]*

*“In the case of written data (e.g. diary), they are difficult to analyse, require enormous expenditure of time on the part of the doctor, risk of error and lack of objectivity of the data” [Italy]*

### 3.2 Relevant digital health design considerations

#### 3.2.1 Interesting functionalities of a digital solution for GHD management

Tailoring the content of the digital health solution was considered an interesting feature to support GHD management. Although both panels agreed on most of the important factors, the Italian panel emphasised the sociocultural level of patients/caregivers, whereas the Korean panel highlighted the character of the individual.

*“We think a lot about the hardware factor but also in terms of the software, we should think about what the contents of the reminder would be and even if the contents are the same, what the nuance is going to be and how to make it more encouraging, whether emojis would be used, etc. All these would be very important factors to consider. Also, we should decide whether it would target the children or the caregivers, and tailor for each patient group.” [Korea]*

*“Instead, for the rest, it is a last point. I would put it’s easy. Transmission, data reliability, ability to maintain data over time. So, these are the three most important things. Cultural, socio-cultural reasons” [Italy]*

Both panels agreed to offer support in the management of GHD through digital health solutions, with the Korean panel contributing to some technical aspects, such as the use of video or artificial intelligence tools (chatbots). They also highlighted the desire to provide just-in-time support.

*“So that’s why I think a chatbot function would be useful because the patients can ask questions to it or ask what to do in certain situations. I used to think that all these questions in a chatbot were answered by human but later, I found out that it wasn’t. So, if such algorithm is developed, this will lessen the workload of HCPs and also enable caregivers to obtain accurate information at the same time.” [Korea]*

Reminders and motivational messages were considered relevant functionalities by both panels; however, the Korean panel discussed more technical details such as frequency or content.

*“While not many actually do as told, I think it still helps them to take their medications regularly. So such alarm or a tool that allows us to check (compliance) would be ideal.” [Korea]*

“Interestingly, if the patient does not take the dose for several days, he sends an alert” [Italy]

Both Korean and Italian experts agreed that providing feedback to patients/caregivers was a relevant functionality. In this regard, both panels identified several technical aspects, with the Korean panel highlighting some advanced features, such as the inclusion of gamification elements. Additionally, this panel pointed out rewards schemes and discussed this topic at length.

*“We say positive feedback. Rewards and positive feedback. The patient sees the smiley faces, he feels good. Quality of life, he is happy” [Italy]*

### 3.3 Perceived benefits and risks of using digital health solutions for adherence monitoring

#### 3.3.1 Healthcare system level

From the point of view of the healthcare system, a few differences were found between the two countries. Although both Korean and Italian panels considered resource optimisation and cost reduction as potential benefits of automatic adherence data collection, the Italian panel perceived that these data could lead to fewer laboratory tests and less drug wastage, whereas the Korean panel focussed on financing issues, especially for insurance-financed treatments.

*“Healthcare system. Less drug waste, therefore economic impact. Who cares then, the only thing, then they would be able to put in more visits if the visits last less, because we are in bad shape. Use number and times because you make fewer visits” [Italy]*

*“Regarding insurance,…, in the case of continuous glucose monitoring, the policy is that the government won’t cover the cost for those with a compliance level of under a certain level. So also with GHD, if the adherence is a lot less than expected from accumulated data, and yet a lot of patients are receiving reimbursement, this might enable changes in reimbursement paradigm.” [Korea]*

The Italian panel felt that automatic adherence data collection would allow them to optimise motivational strategies to encourage patients/caregivers to comply with GHD treatment. Additionally, the panel identified the potential risk of using these data when measurements are not accurate (e.g., dose detection and fake injections).

*“From a therapeutic point of view, I see the consequences only in a positive sense. Obviously, the therapy, maximized clinical efficacy, that’s right. The middle ground: patient support is needed mostly from the HCP and from the national health service. The middle ground is the crisis between the two. Malaise for mistakes, possible? It’s like that, in the sense, it’s not accessible to everyone, because it’s an application. Sharing, how can the patient perceive it? As a task to which he has to obey, let’s say, an added task, a boredom of having to mark things down? A further burden is the last thing, the fact that if the data is not correct there is a risk.” [Italy]*

#### 3.3.2 HCP level

In terms of potential benefits in decision-making, both Italian and Korean HCPs reported that having automatically collected data would allow them to apply more personalised and just-in-time interventions.

*“In this system, would there be a way for us to see patients with bad adherence and give feedback? Usually, those who are good are not the ones that we have to worry about, and the purpose of this (system) is to find those who are in need of help. So, if we could find ways to provide feedback to encourage those with bad adherence, such as providing happy calls, it might be helpful” [Korea]*

The Italian panel perceived some potential inequities because of the lack of digital skills of HCPs, lack of training of HCPs, or lack of non-digital alternatives. Both panels agreed that checking the collected data could lead to increased workload. The Korean panel had a more negative perception, with some of the experts reporting that HCPs could feel guilty for not being able to check collected data as expected by patients/caregivers. Although the Italian panel agreed, they felt that HCPs may even feel more confident because they could use more accurate and reliable data to optimise patient treatments.

*“However, regarding the third question of whether HCPs will welcome using this data, we are already experiencing a high workload and it will immediately be recognised as a burden. So, I think there should be more reward for HCPs than just being able to better take care of their patients. For example, there is a code for a medical fee for provision of CGMs. Likewise, if they could provide a separate code for growth assessment of children receiving GHD treatment, that could be a reward for us for monitoring these patients more closely and going the extra mile for analysis” [Korea]*

*“Because we usually see GHD patients quite frequently for more than 5 years, GHD patients are one of the patient groups that we usually have good rapport with. So, I don’t think we’ve had much issues in terms of sharing data and on the contrary, I think I often felt bad about not giving them enough feedback on the data provided” [Korea]*

*“This here data accuracy, reliable, absolutely certain, objective” [Italy]*

#### 3.3.3 Patient/caregiver level

Both Korean and Italian experts agreed that the use of these digital health solutions helps in prescribing evidence-based therapies that could lead to patient/caregiver empowerment. They linked tracking adherence data to high levels of empowerment.

*“Scenario 3 (the use of Aluetta^®^ with Smartdot™ and the connected digital ecosystem to gather adherence data) the patient’s empowerment and self-efficacy improve markedly. Minimum effort, because the patient has to make a minimum of effort to improve the results. Empowerment when the therapy is followed correctly and the fact that the doctor interacts in real time remotely.” [Italy]*

*“I agree. Of course, there will be a lot more ways and items to be able to link and observe growth curve on the app, which will be beneficial in terms of patient empowerment” [Korea]*

Both Korean and Italian experts believed that automated adherence data collection could influence patient/caregiver motivation and promote adherence. Korean experts agreed that the feedback features would impact the ability of patients/caregivers to self-monitor their GHD and, therefore, improve their self-management. Similarly, Italian experts agreed that the use of digital health solutions can facilitate timely feedback. Some of the experts reported that digital solutions can maximise the opportunities to provide feedback to patients and caregivers. These experts linked feedback to high levels of motivation and satisfaction among children and their families.

*“We have written about the importance of effectiveness in terms of growth, which returns a fairly evident result that brings satisfaction to both the family and the child himself” [Italy]*

*“So, in clinic, regarding health management from the caregiver’s perspective, I think it could give them an impression that their physician is paying attention and caring for them” [Korea]*

Regarding the potential risks associated with the use of digital health solutions, the Italian panel reported that a lack of digital literacy could lead to health inequities. However, the Korean panel considered that a non-digital alternative should be implemented, allowing patients/caregivers to choose their preferred option. Additionally, some of the Korean experts commented on the possibility of patients feeling controlled and potential data privacy issues causing reluctance in data sharing.

*“But even so, patients may prefer the notebook, because using the app would mean disclosing a lot of their private information, which may act as a resistance factor. Writing the values down in a notebook might feel like they are keeping a secret to themselves, while using an app automatically means that the data is accessible by others, which I think will be associated with resistance. So, we need to take into consideration the privacy issue and there should be a way to protect that” [Korea]*

#### 3.3.4 HCP–patient relationship

All experts agreed that automated adherence data collection provides them with accurate and reliable data that they can use to communicate with their patients/caregivers. Automated collection could increase trust between HCPs and patients/caregivers. However, the Korean panel reported that more reliable and accurate data could lead to conflicts between patients and caregivers, which would negatively impact the HCP–patient relationship. They reported that some patients may also be reluctant to come for further visits if HCPs determined that they were lying.

*“As repeatedly mentioned, from management perspective, availability of objective data allows us to build further trust with caregivers. When such tool was not available, I had a diabetes patient whose adherence was really bad despite constantly being told that she needs to improve. So, I gave her an ultimatum by saying that I’d have to transfer her to a different clinic, and she started crying and said that I never encouraged her by saying that she had also been good” [Korea]*

The Italian panel provided mixed opinions. Some experts commented that these objective data could provoke negative feelings of control or intrusiveness among some patients. Conversely, others felt that the HCP–patient relationship may improve because patients/caregivers may feel that HCPs were taking care of them.

*“There are two points: trust and improvement of the doctor-patient relationship and surveillance” [Italy]*

### 3.4 Perceived usefulness and ease of use of Aluetta^®^ with Smartdot™ and the Growzen™ ecosystem

Experts from both countries agreed that Aluetta^®^ with Smartdot™ had a user-friendly format for transforming a pen into a digital health solution. They did not perceive any changes in terms of weight when the Smartdot™ accessory was attached to the Aluetta^®^ pen, making it suitable for use by children.

*“Considering the size of the pen, which is substantial, I think they’ve done their best with the technologies available to minimise the cap size” [Korea]*

Although some Korean experts commented on the desirability of incorporating some form of feedback to indicate that the coupling process has been successfully completed, most of the Italian experts reported that they missed receiving feedback and preferred to receive audio feedback.

*“It would take something like click, which gives the feeling that it engages, in my opinion. It would take a shot when you put it” [Italy]*

Between the two countries, the main difference was highlighted in charging. The Italian panel expressed concerns about battery life, fearing that forgetting to charge the device could result in data loss. Conversely, the Korean panel emphasised the technical aspects of charging, expecting a more advanced process like wireless charging to make it easier and prevent data loss.

*“Lastly, it should be easy to charge the device, for example, adopting a wireless charging system that will automatically charge the device once it’s placed and stored in the case, rather than having to charge every two weeks. So, these are the five suggestions” [Korea]*

Apart from these features, the HCPs from both countries also shared their comments on the perceived ease of use of Aluetta^®^ with Smartdot™, as summarised in **Table 1**, and the Growzen™ digital health ecosystem. The Growzen™ Buddy patient app was considered as an easy-to-use application by experts from both countries.

**Table 1:** Summary of experts’ comments on the perceived usefulness and ease of use of Aluetta^®^ with Smartdot^™^.

Component	Topic	Summary of comments from Italy	Summary of comments from Korea
Aluetta^®^ with Smartdot™	Location of components	The administration button of Aluetta^®^ with Smartdot™ was easier to use and more ergonomic, improving the user experience.	The device was manageable and its dimensions made it suitable for use by both adults and children.
	Pairing and configuration	Aluetta^®^ with Smartdot™ configuration in the Growzen™ Buddy patient app was easier to do than the new smartphone configuration.	The pairing process was found to be similar to that used in other current Bluetooth^®^ devices.
	Ease of use	Aluetta^®^ with Smartdot™ was quite similar to other electronic devices, and people who were familiar with it could easily use it. Aluetta^®^ with Smartdot™ improved the usability of the pen.	Aluetta^®^ with Smartdot™ mounted was lightweight and perfectly suitable for children.
	Reliability and accuracy	–	Aluetta^®^ with Smartdot™ may be helpful in analysing the cause of non-adherence and objectively verify patients with poor adherence by documentation.
	Target users	–	Aluetta^®^ with Smartdot™ will likely be used by new patients and existing adherent patients.
	Perceived risks		Aluetta^®^ with Smartdot™ may have issues with connection due to refrigeration.

*“The application, what do we think? It is objectively easy; you immediately understand how to use it;” [Italy]*

*“Regarding how to attract the existing Aluetta^®^ users, one of the biggest barriers will be how fast they get used to the application. In other words, we have to minimise time wasting that may arise from the app” [Korea]*

The use of the Growzen™ Buddy patient app was perceived to be easy for healthcare professionals to teach and for patients/caregivers to learn.

*“It is easy to explain to an assistant/patient how to configure and associate the device with the APP” [Italy]*

*“Is the setup easy to learn? It’s easy, also because you don’t need to update” [Italy]*

*“I think such app would really help. If we provide thorough explanation at first, it should be useful for patients and caregivers” [Korea]*

Regarding appearance, both Italian and Korean experts found the graphical user interface of the application to be user-friendly and appropriate for patients/caregivers.

*“The colours, the icons, the combination of data; navigation is easy and intuitive, is it clear and understandable? I would say that these 5 are perhaps the most interesting. Even the reminder isn’t bad, but there’s not much to say about the reminder. Does the mobile app help users gain more insight into their adherence behaviour? Yes, of course, that’s why it’s made, so I’d say it’s the most fitting” [Italy]*

*“So maybe the users will be interested in the initial phase, because it kind of looks like a game, but in order to maintain the momentum, then we would need to provide some kind of reward. And while it may depend on the character of the patients, those who show good growth would probably have a high satisfaction level with the application” [Korea]*

Experts from both the Korean and Italian groups commented that the Growzen™ Buddy patient app implemented several feedback strategies that would positively impact patients in their adherence.

*“Is feedback useful when it has been configured? Very helpful” [Italy]*

*“The mobile app helps users to get more information about their joining behaviour. The most important of all” [Italy]*

*“In a sense that this device is about recording the treatment history, I think it could be meaningful for them to see the record of treatment history, And I think this will be positive impact on caregivers” [Korea]*

Italian experts agreed that the Growzen™ Connect HCP platform can be used to engage and discuss reports with patients/caregivers.

*“The platform is our stuff. APP is something mobile, SMS is mobile, we can put the platform just* www.growzenconnect.com. *We put it on reports, it is consulted only by the healthcare provider, therefore only by us. But it is something that obviously serves the patient’s purposes, therefore something digital in any case.” [Italy]*

## 4 Discussion

**Figure 3:**
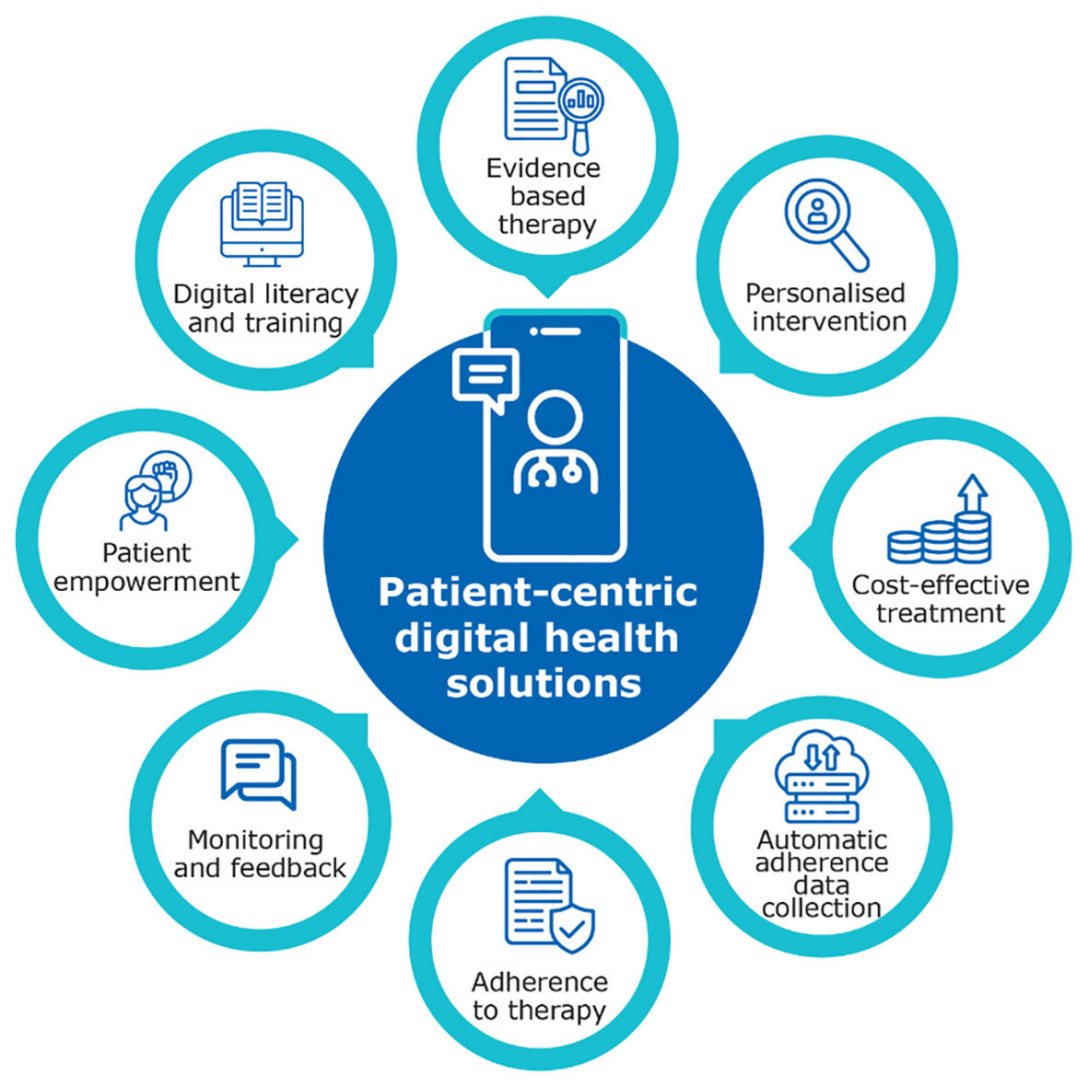
Patient-centric digital health solutions.

This study comprehensively explored HCPs’ perspectives on the adoption of digital health solutions and the acceptance of a digital device ecosystem across Korea and Italy. Understanding the nuances of these perspectives is indispensable for developing strategies to overcome the challenges and leverage the opportunities presented by the ongoing digital transformation in healthcare. Although HCPs appreciate the potential of digital health solutions to improve patient engagement and, hence, clinical outcomes, the participatory workshops revealed several aspects on how this digital transformation is impacting treatment options and the need for digital literacy for successful implementation (**Figure 3**).

The method employed to conduct the participatory workshops facilitated the collection of perspectives of experts from universities and hospitals across Korea and Italy, wherein they shared clinically valuable and understandable technology acceptancy information governing potential barriers and facilitators for the use of Aluetta^®^ with Smartdot™ and the Growzen™ Buddy patient app and the Growzen™ Connect HCP platform. The qualitative analysis compared the opinions of the experts considering two different groups—Italy and Korea—and a researcher reviewed all themes and comments included in them. It was observed that the healthcare systems in both countries are different. As digitalisation was well accepted in Korea given the technical readiness and awareness among patients, HCPs’ adaptation to digital health solutions could be positive. Conversely, the Italian national health system had limited human and technical healthcare resources to support the GH digital health ecosystem, and patients’/caregivers’ adoption of digital health solutions varied depending on an individual’s characteristics, skills, or motivation level. With the spectrum of options available with patients and caregivers, the choice of digital health solution may impact adherence. Therefore, it would be crucial to consider the specific needs and preferences of the patients, caregivers, and HCPs and have features that could be useful to support patients/caregivers in managing their conditions in both countries. The analysis revealed some of the risks and benefits associated with the use of digital solutions for adherence monitoring, such as accessibility to adherence data, data-driven clinical decisions, visibility of results, and strengthened HCP–patient relationships. HCPs from both countries perceived Aluetta^®^ with Smartdot™ as an excellent digital health solution for GH therapy that can create scientific evidence on the relationship between adherence and efficacy.

Over many years, the digital devices for diagnosis, treatment administration, and monitoring have evolved with technological advances in mobile connected health, artificial intelligence, digital patient support programmes, telemedicine, and gamification using virtual and augmented reality (3). To date, the perspectives of HCPs towards digital health solutions have not been studied for paediatric GH therapies using injector pens. This article presents first-of-its-kind insights that emphasise the benefits of the digital ecosystem, constraints of HCPs and the need to address important aspects related to the acceptance of such technology upgrades. Many of the statements from the clinicians reinforced the adherence support described by the World Health Organization, which included elements such as literacy and support (17). Multiple clinicians highlighted the importance of having more adherence data to improve clinical practice and research. This feedback is congruent with recent reviews on the use of sensors to monitor adherence (18, 19).

However, the use of connected sensors for adherence can affect the cost effectiveness of the treatment, which is not always quantified and recognised by healthcare systems. The importance of the value of adherence data appears to be clearly linked to the provision of visualisations to facilitate condition management, which also includes the visualisation of data in both the mobile application and interface for the doctors. There are emerging initiatives on creating standards for adherence reporting that also mention such needs; however, more research is required in that area (20). In the case of connected injection pens, it is essential to consider that the user interfaces encompass not only the connected pen but also the mobile application used for pairing process to link the Aluetta^®^ with Smartdot™ with the Growzen™ Buddy patient app.

All HCPs highlighted that a connected injector device such as Aluetta^®^ with Smartdot™ in the Growzen™ ecosystem can help personalise care by enabling patient empowerment and clinical decision-making. Aluetta^®^ with Smartdot™ was considered to be easy to use, easy to learn and teach, ergonomically suitable for use by both children and adults, comfortable to be transported, robust, easy to charge, and easy to pair with other devices, thereby providing a better administration experience. From HCPs perspective, the Growzen™ Buddy patient app would be easy to use, easy to learn, and the feedback provided by the application would be valuable to motivate patients. Growzen™ Connect HCP platform was considered useful for data analysis by HCPs and for promoting discussion with patients/caregivers. Furthermore, the following aspects were considered actionable: 1) healthcare systems need to include adherence monitoring as part of pharmacoeconomic models considered by payors; 2) training on the use of adherence data derived from connected devices should be promoted to both clinicians and patients; 3) easy-to-use platforms that support HCPs in data analysis should be accessible, including alerts when events requiring attention occur, such as actionable recommendations when a lack of adherence is detected or predicted; 4) the possibility of prediction tools based on newly captured data should be explored to bring about a positive impact on research; 5) digital literacy and privacy concerns experienced by some users should be addressed, and the potential negative impact of using digital health or health disparities should be reduced; and 6) best practices to incorporate such sources of data into the provision of care should be studied, especially considering the impact on clinicians’ time.

One of the challenges observed in the true adoption of a digital health ecosystem is the long-term engagement of HCPs and patients/caregivers with digital health applications/devices. Often owing to the limitations of time or digital literacy, sustained engagement with technology poses a challenge. Sensor-based devices such as Aluetta^®^ with Smartdot™ present with an alternate communication platform that is essential to engage patients/caregivers and develop user-centered solutions for the treatment and management of GHD (21).

The participatory workshops in these two countries examined the perspectives of a small group of experts over a short period. Further studies are required to determine the extent of digital health solution adoption among HCPs and patients/caregivers. Furthermore, with the progression and evolvement of technology, some desired features discussed may be incorporated, and HCPs’ recommendations may be altered. Although these perspectives may not be universal, they do help in the development of an individualised approach to GH treatment.

## 5 Conclusion

HCPs are one of the foremost stakeholders in the implementation of digital health solutions. Our participatory workshop helped capture meaningful insights from them as experts. The main findings highlighted that experts considered/perceived Aluetta^®^ with Smartdot™ within the Growzen™ digital health ecosystem as user-friendly, intuitive, and easy-to-use digital health solutions. Aluetta^®^ with Smartdot™ enabled automatic, real-time injection data transmission to support adherence monitoring and data-driven treatment decisions, thereby helping understand the reasons for suboptimal response or adherence issues with GHD therapy. The availability of unbiased, reliable, and accurate data transmitted by the device would be beneficial and help generate new evidence-based knowledge to support GHD therapy, strengthen patient–HCP relationships, and empower patients throughout the treatment process. The findings from these workshops can further contribute towards novel insights to enable HCPs to better adopt and prescribe digital health solutions as part of their routine care and support researchers with new clinically relevant datasets for better management of GHD.

## Data availability statement

The original contributions presented in the study are included in the article. Further inquiries can be directed to the corresponding authors.

## Ethics statement

All procedures performed in this study were in accordance with European and national ethical guidelines, the European Code of Conduct for Integrity in Research, the Universal Declaration of Human Rights and the Helsinki declaration. Each participant was informed about the research topic and procedures before joining the expert panel. They signed an agreement giving their consent to participate. Participants in this expert panel were not considered human subjects. The main topic of this research was the digitization of healthcare. The experts provided their opinions, based on their experience, on this topic by assuming an advisory role. Therefore, they were not the main subjects of the study. In this study, the experts’ opinions included as quotes in the paper were pseudo- anonymised. No sensitive information was used or collected, and the contributions of the expert panel had no impact on others. In addition, no interventions were made in this study. Therefore, no additional ethical approval was deemed necessary. The studies were conducted in accordance with the local legislation and institutional requirements. The participants provided their written informed consent to participate in this study.

## Author contributions

ORR: Conceptualization, Formal analysis, Investigation, Methodology, Validation, Writing – review & editing. HWC: Conceptualization, Methodology, Validation, Writing – review & editing. MFF: Conceptualization, Methodology, Validation, Writing – review & editing. EV: Conceptualization, Methodology, Validation, Writing – review & editing. CKC: Conceptualization, Methodology, Validation, Writing – review & editing. RDM: Validation, Writing – review & editing, Conceptualization, Methodology. FF: Conceptualization, Methodology, Validation, Writing – review & editing. HSL: Conceptualization, Methodology, Validation, Writing – review & editing. CG: Conceptualization, Methodology, Validation, Writing – review & editing. JK: Conceptualization, Methodology, Validation, Writing – review & editing. AK: Conceptualization, Methodology, Validation, Writing – review & editing. JEM: Conceptualization, Methodology, Validation, Writing – review & editing. MLI: Conceptualization, Methodology, Validation, Writing – review & editing. JY: Conceptualization, Methodology, Validation, Writing – review & editing. AA: Conceptualization, Methodology, Validation, Writing – review & editing. Y-JR: Conceptualization, Methodology, Validation, Writing – review & editing. EK: Conceptualization, Methodology, Validation, Writing – review & editing.
